# Mutagenesis alters sperm swimming velocity in Astyanax cave fish

**DOI:** 10.1038/s41598-022-22486-5

**Published:** 2022-11-15

**Authors:** Richard Borowsky, Haining Chen

**Affiliations:** Department of Biology, New York University, New York, USA

**Keywords:** Evolutionary genetics, Evolutionary theory

## Abstract

We investigated the hypothesis that intra ejaculate sperm competition screens against the transmission of deleterious alleles, including new mutants, from male parent to offspring. Recent investigations have established that sperm haploid genotypes can have major effects on sperm traits such as cellular robustness, longevity, and fertilization success. However, there is no evidence that new mutations can meaningfully affect sperm phenotypes. We tested this directly by comparing sperm from mutagenized and non-mutagenized control males in *Astyanax* fish. We used N-ethyl-N-nitrosourea (ENU) to induce single base substitutions in spermatogonial stem cells. We looked at swimming velocity, an important factor contributing to fertilization success, and flagellar length. Variability in swimming velocity was significantly higher in sperm from mutagenized males than in control sperm, reflecting their increased allelic diversity. In contrast, flagellar length, which is fixed during diploid stages of spermatogenesis, was unaffected by ENU treatment. We briefly discuss the implications of intra-ejaculate screening for maintenance of anisogamy and for outcomes of assisted reproductive technology.

## Introduction

A typical vertebrate ejaculate contains millions of sperm cells, but only a small number are needed to fertilize the available ova. There is no accepted explanation for this apparent functional redundancy that applies to every species. It makes sense in polyandrous species, where inter-ejaculate sperm competition puts a premium on each male’s contributing as many sperm as possible^1^. But why does this occur in monandrous species? Here there is no similar benefit in producing large numbers of sperm per ejaculate, as all the sperm have been produced by the same male. Nevertheless there still is competition among sperm within the same ejaculate, and, as a result of meiosis, there is always genotypic variability among them. Thus, the outcomes of competition may be influenced by the haploid genotypes of the individual spermatozoa if they are expressed phenotypically^2,3^, and sib sperm competition may screen out those carrying detrimental alleles in their haploid genomes.

Modeling the outcomes of sib sperm competition led to an important discovery: In competition between two equally abundant genotypes of sib sperm, where one type has a small advantage over the other, the marginally better phenotype virtually always wins^4^. This is so even when the advantage is very small, like a 1% faster swimming velocity. This near inevitability is due to the huge numbers of competing cells and their individual infinitesimal probabilities of success.

If marginally worse phenotypes nearly always fail to fertilize the ovum, even when well represented in the ejaculate, then a single sperm bearing a unique detrimental allele would, at first glance, seem to have no chance to be a fertilizing gamete. Nevertheless, the common observation is that detrimental alleles are indeed transmitted during fertilization at or near Mendelian expectations. An explanation for this may be that in a real fertilization, alleles at many loci are simultaneously in play and detrimental alleles may hitchhike with favorable ones.

We suggest that sib sperm competition may serve as an imperfect but still consequential screen limiting the transmission of deleterious new mutations to a zygote. If so, several conditions must be met. First, at least some sperm phenotypes must be determined by their haploid genotypes. Recent work demonstrates this does occur^2,3,5^. Second, alleles phenotypically expressed in the haploid stage and subject to selection via sib sperm competition should also have phenotypic consequence after fertilization, in the zygote. This has been demonstrated for sperm longevity and swimming behavior, which are correlated with zygotic growth rates and fitness^5–7^. Third, the phenotypic value of alleles expressed in sperm would have to be correlated with their phenotypic value in the zygote. That is, alleles deleterious (or advantageous) in the sperm would be deleterious (or advantageous) in the zygote^8^. If these conditions hold, selection via sib sperm competition could speed evolution, because mutations, which are most often recessive, could be selected for in the haploid sperm before they are introduced into a population, where their effects would be hidden for generations in the diploid state.

As the literature does not reveal whether or not new mutations are expressed in sperm during the sib sperm competition stage, we conducted several experiments to shed light on the matter. We tested the effects of the mutagen N-ethyl-N-nitrosourea (ENU) on the sperm of *Astyanax mexicanus* males. ENU treatment in fishes mainly produces recessive point mutations in the sperm stem cells^9,10^. Thus, most of the new mutations could not be expressed in the diploid state, but might be expressed in the haploid sperm. Since each mutation in a stem cell is confined to the descendants of that stem cell, there would be relatively few copies of specific new mutations in the ejaculate. However, their aggregate effects could be detected if each sperm cell received many mutations, as is the case with ENU mutagenesis^9^. It is not possible to predict exactly how a new mutant would affect continuous traits like swimming speed or flagellar length. But it is reasonable to predict that increased genetic diversity should cause increased trait variability.

We analyzed sperm behavior using computer assisted sperm analysis (CASA)^11,12^. Based on tracking records CASA calculates several sperm swimming phenotypes, including curvilinear velocity (VCL), the average velocity of a sperm cell along its actual path. Two other CASA phenotypes are VAP, the velocity measured along a smoothed path, and VSL, which is based on the straight-line distance between start and finish positions. Thus, both VAP and VSL are functions of path curvature as well as velocity. CASA also calculates four other phenotypes designed to highlight path components such as linearity, progressivity, and swimming efficiency. Each of these metrics incorporates one or more of the velocity indices as divisors, thus increasing the variances of their estimates and reducing their power in statistical analyses. As the most straightforward of the CASA phenotypes, we focused on VCL.

We compared two sperm phenotypes in ENU treated and untreated control males: VCL and flagellar length. Swimming velocity is an important factor contributing to fertilization success in fishes^13^, and flagellar length is considered to be a potential determinant of swimming velocity in externally fertilizing species^14^. VCL is a sperm phenotype exhibited by mature haploid sperm, whereas flagellar length is a phenotype that develops before the sperm are mature, when the sister spermatids are still syncytial and functionally diploid^15^. Thus, our prediction was that, within an ejaculate, VCL would be more variable in mutagenized than in non-mutagenized males, but that flagellar lengths would not vary significantly between the two groups.

## Results

### Experiment 1

**Figure 1:**
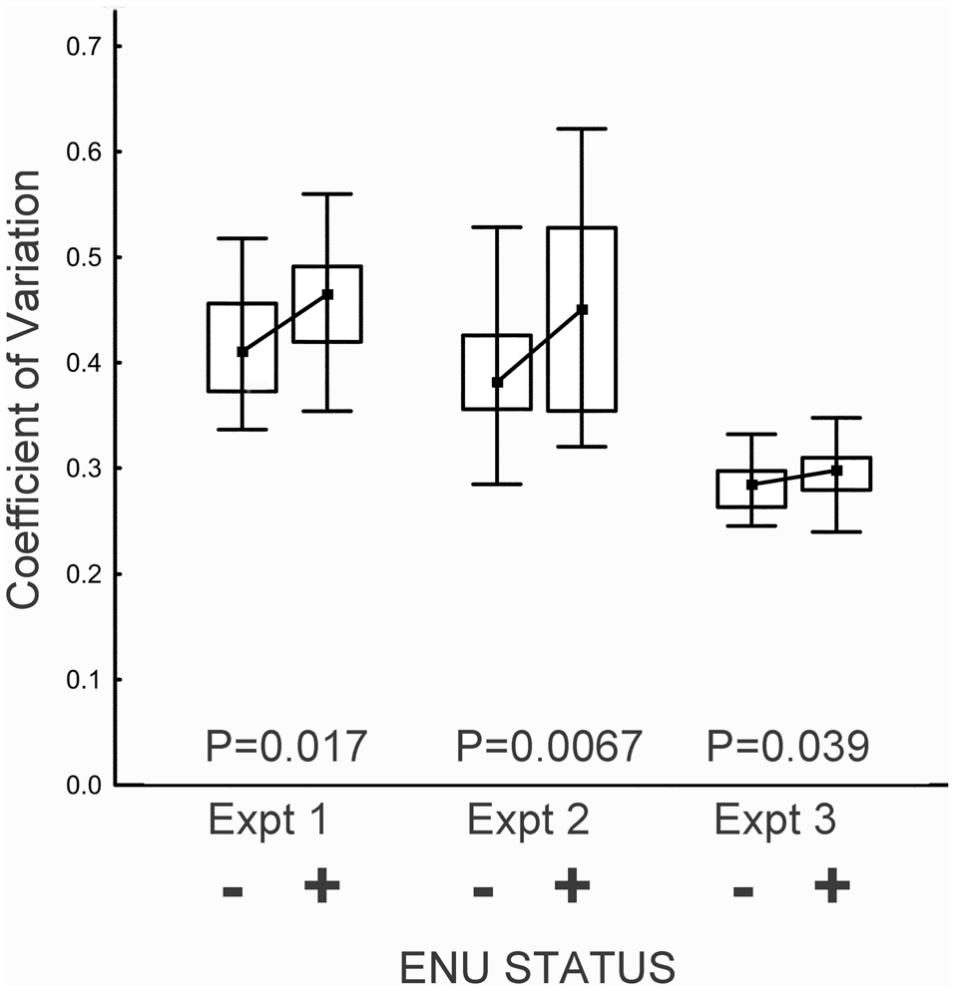
Variability of VCL within ejaculates is significantly greater in sperm from ENU treated males (ENU +) than from control males (ENU −). The inner box marks the median, the outer box spans the 25th through 75th percentiles, and the whiskers mark the range.

We quantified curvilinear velocity (VCL) of individual sperm cells from mutagenized and non-mutagenized males using CASA (Material and Methods). For each male, we calculated the Coefficient of Variation (CV) of VCL among its tracked sperm. As predicted, the variability of VCL was greater for sperm from mutagenized males than for sperm from control males (Mean CV: 0.457 vs. 0.414, t_31_ = 2.21, one tailed *p* = 0.017; Fig. 1, Table 1).

**Table 1:** Variability of VCL within ejaculates differs significantly between ENU treated males and control males. N_0_ and N_1_ are sample sizes.

	ENU -	ENU +	Effect			One-Tailed	ENU -	ENU +
	Mean CV	Mean CV	Size %	t value	df	*p*	N_0_	N_1_
Expt 1	0.414	0.457	10.4	2.21	31	0.017	19	14
Expt 2	0.391	0.453	15.9	2.58	43	0.0067	29	16
Expt 3	0.283	0.297	4.9	1.81	42	0.039	24	20

### Experiment 2

We repeated Experiment 1, essentially obtaining the same result. Average CV was again greater in mutagenized versus control sperm (0.453 vs. 0.391, t_45_ = 2.58, one-tailed *p* = 0.0067; Fig. 1, Table 1).

### Experiment 3

We modified procedures (as detailed in Material and Methods) to reduce crowding in the videos to be analyzed by CASA. The principal effect of the modifications was to reduce the overall variability in the data (Fig. 1). As in Experiments 1 and 2, the variability in VCL of sperm from ENU treated males was greater than that of sperm from control males (CV of 0.297 vs 0.283, t_42_ = 1.81, one tailed *p* = 0.039, Fig. 1, Table1). Raw data for experiments 1–3 are in Supplementary Files 1, 2, 3.

### Experiment 4

To test whether flagellar lengths are affected by mutagenesis, we obtained sperm from 38 males (18 ENU treated and 20 untreated controls) and measured their tail lengths (average sample size > 50). The two groups did not differ in variability of tail length (CV = 0.140 vs. 0.137, t_36_ = 0.51, *p* = 0.61) or in average tail length; 16.75 versus 17.2 μ, t_36_ = 1.58, *p* = 0.124.

To test whether flagellar length is a determining factor for swimming velocity in this system, we measured tail lengths of treated and control males tested for VCL in Experiment 1. There was no significant relationship between tail length and velocity (r =  − 0.10, n = 47) nor was there a significant correlation of CV of tail length with ENU treatment status (r = 0.13, n = 47). Thus, the differences among treatments we see in VCL are unlikely due to differences in flagellar lengths. Raw data are in Supplementary Files 4 and 5.

We tested the six other CASA phenotypes for any potential correlations with ENU treatment. With two exceptions, there was little significant. VAP and VSL, two other velocity phenotypes, were significantly correlated with ENU treatment in Experiment 1. For VAP CV of 0.639 versus 0.555, t_32_ = 2.83, one tailed *p* = 0.012 and for VSL CV of 0.674 versus 0.591, t_32_ = 2.42, one tailed *p* = 0.020. Raw data are in Supplementary Files 1, 2, 3.

## Discussion and conclusions

Both predictions made for the effects of increased mutation rate on sperm phenotype were supported by the data. The first was that treatment with ENU would have no effect on flagellar length and its variability. Because flagellar length is determined at a diploid stage, ENU induced mutations, overwhelmingly recessive^9^, should have little phenotypic consequence. As predicted, the data revealed no apparent correlations.

We also predicted that mutagenesis by ENU would increase the variability of sperm swimming velocity (VCL), a sperm phenotype exhibited post-meiotically. The results of experiments 1–3 support this prediction and the pattern is consistent with cumulative small effects of ENU mutations on VCL at the level of haploid sperm.

These results show that new mutations can affect a sperm phenotype critical to fertilization success and that sib sperm competition could serve as a screen against the passage of alleles with deleterious effects. What might be the correlates and consequences of such a screen?

Sperm cells are unique among cell types not only in their great abundance, noted previously, but because large proportions of them are non-functional. Within a single ejaculate, sperm may exhibit enormous variation in morphology and behavior, including missing or supernumerary flagella, two heads, lack of midpiece, and lack of motility^16^. These extreme variants are widely believed to be incapable of fertilizing an ovum^17^. Even among sperm that are motile and appear normal in morphology, there are large differences in swimming speed and trajectory which likely influence the probabilities of successful fertilization^18^. High proportions of morphologically abnormal sperm cells have been documented from a broad range of animal species, including humans^16,17,19–25^. The phenomenon is so widespread that it is considered to be a “normal” condition of sperm cell populations. For example, a World Health Organization study on sperm characteristics of fertile men found that the median percentage of their sperm failing to exhibit progressive motility was 45% and that the median percentage of sperm with abnormal morphologies was 85%^26^. It is worth emphasizing that these statistics are for men of “normal” fertility.

Why do ejaculates generally contain such high proportions of aberrant sperm cells? One suggestion is that sperm cells are so complex in form and function that their manufacture, free of flaw, may be difficult. In this view, the non-functional sperm are defective products that have escaped quality control^18^. In contrast, we suggest that abnormal sperm cells are actually key components of an adaptation, which is the proposed screen against the passage of deleterious alleles into the next generation^2^. If alleles that are deleterious in sperm are also deleterious in the zygote, then eliminating them before the possibility of fertilization should increase the fitness of zygotes that do occur^2,3,8^. We suggest that sperm cells are inherently shoddy in construction and thus easily pushed over to non-functionality by the deleterious alleles they carry. That is, a basic shoddy construction is the adaptation. The adaptive advantage of producing poorly constructed sperm is that they could be sensitive to their haploid genomes. Thus, if their haploid genetics were substandard, the cells would be selected against prior to fertilization.

An unanswered question is how the haploid genotypes of sperm cells could be expressed. DNA in the spermatozoon is compacted and largely transcriptionally inactive. At an earlier stage, haploid spermatids are connected by cytoplasmic bridges that can allow mixing of mRNA contents^15^. However, single cell transcriptomics shows this homogenization is incomplete^27^. Thus, there are allelic biases in mRNA content among sisters that allow for differential expression. Recent work shows that sperm haploid genetics is correlated with various sperm phenotypes including swimming speed, longevity and resistance to chemical challenge, a proxy for robustness^2,3,5–7^, so a haploid screen is plausible.

If further study shows that at least some of the same mutations that are detrimental to the individual in the diploid form are also harmful to sperm in the haploid form, then these mutations would tend to be eliminated from competition for the egg before fertilization occurred; this would constitute virtually load free selection, and the more sperm competing, the lower the selectional load.

Our results warrant further study because of their broader implications for human health and evolutionary theory. Studies show that children conceived by assisted reproductive techniques (ART) have a 40% greater risk of developmental abnormalities than children conceived naturally^28–30^. Although the reasons for this are debated, it is important to note that sperm competition is largely or completely eliminated in conceptions resulting from ART. Bypass of the screen may explain the increased developmental risks. New artificial screens, devised to better mimic the natural environment, may prove effective in minimizing the passage of bad alleles via ART^31^.

Anisogamy is maintained in the face of the two-fold cost of sexual reproduction^32^; the selective forces that insure this are debated^33^. We suggest that the key is the small size of the sperm which facilitates their deployment in vast numbers^34^. It is this large number of sperm that allows effective screening that is essentially load free^4^. While there are tradeoffs between size and number of sperm cells^1^, any mutation acting in the diploid phase that decreased the number of sperm would decrease the effectiveness of the screen and be a target for selective elimination.

## Materials and methods

### Ethics statements

Fish were cared for under Protocol #05–1235 approved by the New York University Animal Welfare Committee (UAWC). All methods were performed in accordance with the relevant guidelines and regulations. The study is being reported in accordance with ARRIVE guidelines. The authors have no competing interests.

### Statistics

Statistical analyses were done using Statistica (Tibco). We predicted that ENU treatment would increase the variability of VCL and thus we calculated one-tailed probabilities. Probabilities calculated for testing hypotheses of flagellar length were two-tailed. The outlier screening function of Statistica was used to identify outliers in the raw data, which were then excluded from further analysis.

### Stocks

We used commercially sourced *Astyanax mexicanus* cave fish for these experiments because they are inbred, having been maintained in captivity for numerous generations since their original collection in 1936^35^. They were approximately 1.5 years old when mutagenized and 2.5 years old at the start of the experiments.

### Mutagenesis

Mature males were bathed in a solution of system water containing 2.5 mM N-ethyl-N-nitrosourea (ENU), following published protocols^9^. Thirty-eight males were initially treated for one hour. There was 16% mortality during the treatment. After five weeks recovery time, the remaining 32 males were treated a second time for 25 min. Twenty-nine of the 32 survived (9% mortality). The fish were then set aside for a full year prior to commencement of the present experiments. In *Danio*, the mutagenic effect of ENU treatment is roughly proportional to the product of exposure concentrations and time^9^. Thus, we quantified the mutagenized males’ exposure to ENU in the present experiment as approximately 3.5 mM hours.

The effectiveness of ENU treatment in this experiment can be estimated from published data on other freshwater fishes. Sequencing data on three species of Lake Malawi cichlids allowed estimation of spontaneous mutation rates of 3.5 × 10E-9 per base per generation^36^, which we took as a reasonable estimate for the unknown rates in *Danio* and *Astyanax*. In *Danio*, a study of ENU induced mutations (exposure of 15 mM hours) in four pigmentation genes gave an estimate of a per locus mutation rate of 1.1 × 10E-3^9^. The summed coding sequence of these genes (*i.e.*, the mutational target size) was estimated as 9189 bases. An exact figure is unknown because one gene used in^9^, “brass,” has not yet been identified in the *Danio* genome. Its target size was taken as the average of the other three markers (*SLC24A5, Kita*, and *Oca2*). Adjusting for the difference in ENU exposure and for an estimated proportion of synonymous mutations of 0.35^37,38^, the ENU induced mutation rate in our experiments is estimated to have been 12.5 times the spontaneous rate. This figure is an underestimate because not all non-synonymous mutations in^9^ would be expected to have observable phenotypes. While rough, the estimate is instructive and indicates that the ENU treatment in this study led to a substantial increase in numbers of new mutations.

### Preparatory treatment and sperm collection

Prior to testing, mutagenized and non-mutagenized males were housed in two 57 L aquaria adjacent to one another on an aquarium rack. Both holding tanks had bubblers operated from the same air pump, and were flow-through in design and fed from the same water source. Illumination and temperature (21 ± 1 °C) were equal in both tanks. For testing, small numbers of individuals (3–6) were transferred out of the holding tanks and into two 21 L treatment tanks adjacent to one another on a nearby rack. They were initially at the same temperature as the holding tanks. Water flow was cut to the treatment tanks and 25 Watt heaters were turned on in each. Over the course of several hours the tanks warmed to 26 ± 1 °C. Final temperatures in the two tanks were close, but not identical, reflecting small differences in the settings of the heaters. To minimize any systematic effects of differences in temperature, the heaters were switched between treatment tanks at each round of experimentation.

The males were maintained at elevated temperature overnight to stimulate production of new sperm for harvest^39^. For sperm collection, the males were anesthetized using 0.025% tricaine-methanesulfonate (MS-222, Western Chemical) for about 60 s. They were then blotted dry, placed on their backs on a wet pad of nylon wool, the gonopore areas were lavaged with Hanks buffered saline solution (HBSS) to suppress activation of the sperm^40^. The subjects were gently squeezed to expel the sperm, samples of which were collected using fine tipped transfer pipets. Samples were placed on ice and used within an hour of collection, either for behavioral study and CASA analyses, or for the measurement of sperm flagellar length.

### CASA analyses

For the CASA experiments we visualized the sperm using an Olympus IMT-2 microscope, recording swimming behavior with negative phase-contrast illumination at 100 frames per second. We used a monochrome camera with resolution of 1280 × 860 pixels (Chameleon3-U3-13Y3M-CS) and FlyCap2 Viewer version 2.13.3.61, (www.flir.com). For experiments 1 and 2 we activated the sperm by mixing 1 μL of sperm suspension with 7 μL of system water. Following activation, 1 μL of well-mixed activated sperm was pipetted into a single well of a 12-well Multi-Test Slide (MP Biomedicals, Irvine, CA, USA), and covered by a coverslip^11,12^. Each sample was tested twice. The median numbers of motile sperm tracked in the two experiments were 113 and 163. The objective used was 20X and the planar eyepiece for the camera was an NFK 3.3.

Based on the results of experiments 1 and 2, we diluted the sperm suspensions for experiment 3 by the addition of small volumes of HBSS prior to recording. This reduced track overlap and collisions. To retain sufficient sample size we increased the number of replicates for each sample. The median number of samples processed per male in experiment 3 was nine and the median number of motile sperm per recording was 50. The objective used was 10X. The change in power from 20 to 10X had two useful effects. First, the field had four times the area in experiment 3 than experiments 1 and 2. Second, the 10X had an increased depth of field, and imaged objects more clearly. The result of all the changes was that we tracked more sperm per male in experiment 3 than in experiments 1 and 2, but had much less crowding, by a factor of eight or greater.

For all three experiments we analyzed the 100 frames starting at 20 s post-activation. CASA identifies individual sperm moving from one frame to the next and reports out several swimming phenotypes^11,12^. Three phenotypes are velocity measures, all of which are highly correlated with one another. Four other phenotypes are derived mainly from the original velocity measures. We focused on the most straightforward measure, curvilinear velocity (VCL). VCL is the average velocity during the tracked time interval, e.g., time divided by total distanced traveled.

### Sperm flagellar measurements

To measure the tail lengths of individual sperm cells, immobilized sperm samples were diluted with appropriate volumes of HBSS to obtain uncrowded images. Then 1 μL of diluted sperm was pipetted into a single well of a 12-well Multi-Test Slide (MP Biomedicals, Irvine, CA, USA), and covered by a coverslip. A Zeiss microscope (Axio, 40X objective) was used for visualizing the structure of sperms, including both sperm head and flagella. Images were processed by FIJI software (NIH), and 50 or more sperm were randomly picked from each male for their flagellum length measurements. Sperm were photographed using a 1.3 MP Monochrome camera (CMLN-13S2M-CS, 1296 × 964 res, FLIR Inc.) and FlyCap2 software (FLIR Inc.).

## Supplementary Information

Supplementary Information 1.

Supplementary Information 2.

Supplementary Information 3.

Supplementary Information 4.

Supplementary Information 5.

## Data Availability

The supplementary information section includes spreadsheets with the raw data usind in computations.

## References

[1] Parker GA (1982). Why are there so many tiny sperm—sperm competition and the maintenance of 2 sexes. J. Theor. Biol..

[2] Borowsky R, Luk A, He X, Kim RS (2018). Unique sperm haplotypes are associated with phenotypically different sperm subpopulations in *Astyanax* fish. BMC Biol..

[3] Borowsky R, Luk A, Kim RS (2019). Sperm swimming behaviors are correlated with sperm haploid genetic variability in the Mexican tetra, *Astyanax mexicanus*. PLoS ONE.

[4] Ezawa K, Innan H (2013). Competition between the sperm of a single male can increase the evolutionary rate of haploid expressed genes. Genetics.

[5] Alavioon G, Garcia AC, LeChatelier M, Maklakov AA, Immler S (2019). Selection for longer lived sperm within ejaculate reduces reproductive ageing in offspring. Evol. Lett..

[6] Alavioon G (2017). Haploid selection within a single ejaculate increases offspring fitness. Proc. Natl. Acad. Sci. U.S.A..

[7] Immler S, Hotzy C, Alavioon G, Petersson E, Arnqvist G (2014). Sperm variation within a single ejaculate affects offspring development in Atlantic salmon. Biol. Let..

[8] Immler S, Otto SP (2018). The evolutionary consequences of selection at the haploid gametic stage. Am. Nat..

[9] Solnica-Krezel L, Schier AF, Driever W (1994). Efficient recovery of enu-induced mutations from the zebrafish germline. Genetics.

[10] Wilkie AOM (1994). The molecular-basis of genetic dominance. J. Med. Genet..

[11] Wilson-Leedy, J. G. & Ingermann, R. L. Computer assisted sperm analyzer. https://imagej.nih.gov/ij/plugins/casa.html (2006).

[12] Wilson-Leedy JG, Ingermann RL (2007). Development of a novel CASA system based on open source software for characterization of zebrafish sperm motility parameters. Theriogenology.

[13] Gage MJ (2004). Spermatozoal traits and sperm competition in Atlantic salmon: Relative sperm velocity is the primary determinant of fertilization success. Curr. Biol..

[14] Simpson JL, Humphries S, Evans JP, Simmons LW, Fitzpatrick JL (2014). Relationships between sperm length and speed differ among three internally and three externally fertilizing species. Evolution.

[15] Braun RE, Behringer RR, Peschon JJ, Brinster RL, Palmiter RD (1989). Genetically haploid spermatids are phenotypically diploid. Nature.

[16] Pitnick S, Hosken DJ, Birkhead TR, Birkhead T, Hosken DJ, Pitnick S (2009). Sperm Biology, an Evolutionary Perspective Ch. 3.

[17] Auger J, Jouannet P, Eustache F (2016). Another look at human sperm morphology. Hum Reprod.

[18] Pizzari T, Parker GA (2009). Sperm competition and sperm phenotype. Sperm Biology: An Evolutionary Perspective.

[19] Garcia-Vazquez FA (2015). Morphological study of boar sperm during their passage through the female genital tract. J. Reprod. Dev..

[20] Garcia-Vazquez FA (2015). Morphometry of boar sperm head and flagellum in semen backflow after insemination. Theriogenology.

[21] Koziol K, Koziorowski M (2015). Morphological defects of epididymal spermatozoa in male roe deer (Capreolus capreolus) during the reproductive season. Pol. J. Vet. Sci..

[22] McPherson FJ, Nielsen SG, Chenoweth PJ (2014). Semen effects on insemination outcomes in sows. Anim. Reprod. Sci..

[23] Opatova P (2016). Inbreeding depression of sperm traits in the zebra finch *Taeniopygia guttata*. Ecol. Evol..

[24] Orido Y (1988). Ultrastructure of spermatozoa of the lung fluke, *Paragonimus ohirai* (Trematoda: Troglotrematidae), in the seminal receptacle. J Morphol.

[25] Stewart KA, Wang R, Montgomerie R (2016). Extensive variation in sperm morphology in a frog with no sperm competition. BMC Evol. Biol..

[26] Cooper TG (2010). World health organization reference values for human semen characteristics. Hum. Reprod. Update.

[27] Bhutani K (2021). Widespread haploid-biased gene expression enables sperm-level natural selection. Science.

[28] Alukal JP, Lamb DJ (2008). Intracytoplasmic sperm injection (ICSI)—What are the risks?. Urol. Clin. N. Am..

[29] Hansen M, Bower C, Milne E, de Klerk N, Kurinczuk JJ (2005). Assisted reproductive technologies and the risk of birth defects—a systematic review. Hum. Reprod..

[30] Hansen M, Kurinczuk JJ, Bower C, Webb S (2002). The risk of major birth defects after intracytoplasmic sperm injection and in vitro fertilization. N. Engl. J. Med..

[31] Suarez SS, Pacey AA (2006). Sperm transport in the female reproductive tract. Hum. Reprod. Update.

[32] Maynard Smith J (1978). The evolution of sex.

[33] Monro K, Marshall DJ (2016). Unravelling anisogamy: Egg size and ejaculate size mediate selection on morphology in free-swimming sperm. Proc. R. Soc. B Biol. Sci..

[34] Parker GA, Begon ME (1993). Sperm competition games—sperm size and number under gametic control. Proc. R. Soc. B Biol. Sci..

[35] Elliot W, Keene A, Yoshizawa M, McGaugh S (2016). Biology and Evolution of the Mexican Cavefish Ch. 3.

[36] Malinsky M (2018). Whole-genome sequences of Malawi cichlids reveal multiple radiations interconnected by gene flow. Nat. Ecol. Evol..

[37] Keightley PD (2009). Analysis of the genome sequences of three *Drosophila melanogaster* spontaneous mutation accumulation lines. Genome Res..

[38] Ossowski S (2010). The rate and molecular spectrum of spontaneous mutations in Arabidopsis thaliana. Science.

[39] Borowsky R (2009). Emerging Model Organisms, A Laboratory Manual, Ch. 19.

[40] Westerfield M (1993). The zebrafish book: A guide for the laboratory use of zebrafish (Brachydanio rerio).

